# Creatine in Health and Disease

**DOI:** 10.3390/nu13020447

**Published:** 2021-01-29

**Authors:** Richard B. Kreider, Jeffery R. Stout

**Affiliations:** 1 Human Clinical Research Facility, Exercise & Sport Nutrition Lab, Department of Health & Kinesiology, Texas A&M University, College Station, TX 77843, USA; 2 Physiology of Work and Exercise Response (POWER) Laboratory, Institute of Exercise Physiology and Rehabilitation Science, School of Kinesiology and Physical Therapy, University of Central Florida, 12494 University Blvd., Orlando, FL 32816, USA; Jeffrey.Stout@ucf.edu

**Keywords:** ergogenic aids, cellular metabolism, phosphagens, sarcopenia, cognition, diabetes, creatine synthesis deficiencies, concussion, traumatic brain injury, spinal cord injury, muscle atrophy, rehabilitation, pregnancy, immunity, anti-inflammatory, antioxidant, anticancer

## Abstract

Although creatine has been mostly studied as an ergogenic aid for exercise, training, and sport, several health and potential therapeutic benefits have been reported. This is because creatine plays a critical role in cellular metabolism, particularly during metabolically stressed states, and limitations in the ability to transport and/or store creatine can impair metabolism. Moreover, increasing availability of creatine in tissue may enhance cellular metabolism and thereby lessen the severity of injury and/or disease conditions, particularly when oxygen availability is compromised. This systematic review assesses the peer-reviewed scientific and medical evidence related to creatine’s role in promoting general health as we age and how creatine supplementation has been used as a nutritional strategy to help individuals recover from injury and/or manage chronic disease. Additionally, it provides reasonable conclusions about the role of creatine on health and disease based on current scientific evidence. Based on this analysis, it can be concluded that creatine supplementation has several health and therapeutic benefits throughout the lifespan.

## 1. Introduction

Creatine supplementation is one of the most studied and effective ergogenic aids for athletes [1]. The multifaceted mechanisms by which creatine exerts its beneficial effect include increasing anaerobic energy capacity, decreasing protein breakdown, leading to increased muscle mass and physical performance [1]. While these well-recognized creatine effects benefit the athlete, creatine may also serve as a potential clinical and therapeutic supplementary treatment to conventional medical interventions [2,3,4,5,6,7,8,9,10]. In this regard, over recent years, researchers have been investigating the potential therapeutic role of creatine supplementation on health-related conditions such as diabetes [11], sarcopenia [4,6,12,13], osteoporosis [2,14], cancer [10,15,16,17,18], rehabilitation [4,19,20,21,22,23,24,25,26], cognition [3,27,28,29], and cardiovascular health [5,6,8,30,31,32], among others. This work has increased interest in creatine use as a nutritional strategy to help maintain functional and mental capacity and, as we age, reduce risk to chronic disease, and/or serve as an adjunctive intervention to help manage disease and/or promote recovery. This special issue aims to provide comprehensive reviews of the role of creatine in health and clinical disease. To do so, we have invited a number of top creatine scholars to contribute comprehensive reviews as well as encouraged colleagues to submit meta-analyses and original research to this special issue. 

As an introduction about creatine’s potential role in health and disease, the following provides a general overview of creatine’s metabolic role, purported benefits throughout the lifespan, and potential therapeutic applications. Additionally, we provide reasonable conclusions about the state of the science on creatine supplementation. This overview will be accompanied by separate, more comprehensive, literature reviews on the metabolic basis of creatine in health and disease as well as the potential role of creatine in pregnancy; children and adolescents; exercise and performance; physical therapy and rehabilitation; women’s health; aging, sarcopenia, and osteoporosis; brain neuroprotection and function; immunity, cancer protection and management; heart and muscle health; and, chronic and post-viral fatigue. We hope that this review and special issue will help readers and medical practitioners better understand the safety and efficacy of creatine supplementation in a variety of populations and provide recommendations about future research needs.

## 2. Methods

A systematic review of the scientific and medical literature was conducted to assess the state of the science related to creatine supplementation on metabolism, performance, health, and disease management. This was accomplished by doing keyword searches related to creatine supplementation on each topic summarized using the National Institutes for Health National Library of Medicine PubMed.gov search engine. A total of 1322 articles were reviewed with relevant research highlighted in this systematic review.

## 3. Metabolic Role

Creatine (*N*-aminoiminomethyl-*N*-methyl glycine) is a naturally occurring and nitrogen-containing compound comprised from amino acids that is classified within the family of guanidine phosphagens [1,33]. Creatine is synthesized endogenously from arginine and glycine by arginine glycine amidinotransferase (AGAT) to guanidinoacetate (GAA). The GAA is then methylated by the enzyme guanidinoacetate N-methyltransferase (GAMT) with S-adenosyl methionine (SAMe) to form creatine [34]. The kidney, pancreas, liver, and some regions in the brain contain AGAT with most GAA formed in the kidney and converted by GMAT to creatine in the liver [35,36,37]. Endogenous creatine synthesis provides about half of the daily need for creatine [35]. The remaining amount of creatine needed to maintain normal tissue levels of creatine is obtained in the diet primarily from red meat and fish [38,39,40,41] or dietary supplements [1,42,43]. About 95% of creatine is stored in muscle with the remaining amount found in other tissues, like the heart, brain, and testes [44,45]. Of this, about 2/3 of creatine is bound with inorganic phosphate (Pi) and stored as phosphocreatine (PCr) with the remainder stored as free creatine (Cr). The total creatine pool (Cr + PCr) is about 120 mmol/kg of dry muscle mass for a 70 kg individual who maintains a diet that includes red meat and fish. Vegetarians have been reported to have muscle creatine and PCr stores about 20–30% lower than non-vegetarians [46,47]. The body breaks down about 1–2% of creatine in the muscle per day into creatinine which is excreted in the urine [46,48,49]. Degradation of creatine to creatinine is greater in individuals with larger muscle mass and individuals with higher physical activity levels. Therefore, a normal-sized individual may need to consume 2–3 g/day of creatine to maintain normal creatine stores depending on diet, muscle mass, and physical activity levels. In fact, Wallimann and colleagues [50] noted that since creatine stores are not fully saturated on vegan or normal omnivore diets that generally provide 0 or 0.75–1.5 g/day of creatine, daily dietary creatine needs may be in the order of 2–4 g/person/day to promote general health [1,50]. The most effective and rapid way to increase muscle creatine stores is to ingest 5 g of creatine monohydrate four times daily for 5–7 days (i.e., 0.3 g/kg/day) [46,49]. However, some studies have shown that consuming 2–3 g/day of creatine for 30 days can also effectively increase muscle creatine stores [46,49]. Dietary supplementation of 20–30 g/day of creatine monohydrate for up to 5 years has also been studied in some clinical populations who need higher levels to increase brain concentrations of creatine, offset creatine synthesis deficiencies, or influence disease states [51,52,53].

Creatine and phosphagens play a critical role in providing energy through the creatine kinase (CK) and PCr system [50,54,55]. In this regard, the free energy yielded from the enzymatic degradation of adenosine triphosphate (ATP) into adenosine diphosphate (ADP) and Pi by CK serves as a primary fuel to replenish ATP for cellular metabolism. Breaking down PCr into Pi and Cr with the enzyme CK yields about 10.3 kcals of free energy that can be used to resynthesize ADP -+ Pi into ATP [38,39,56,57]. The ability to replenish depleted ATP levels during high-energy demand states like intense exercise or in conditions where energy production is either impaired (e.g., ischemia, hypoxia) or insufficient due to increased demand (e.g., mental fatigue, some disease states) is important in maintaining ATP availability.

Creatine enters the cytosol through creatine transporters (CRTR) [58,59,60,61]. In the cytosol, creatine and associated cytosolic and glycolytic CK isoforms help maintain glycolytic ATP levels, the cytosolic ATP/ADP ratio, and cytosolic ATP-consumption [50]. Additionally, creatine diffuses into the mitochondria and couples with ATP produced from oxidative phosphorylation and the adenine nucleotide translocator (ANT) via mitochondrial CK. PCr then diffuse back into the cytosol and help meet energy needs. This coupling reduces the formation of reactive oxygen species (ROS) and therefore creatine acts as a direct and/or indirect antioxidant [18,21,62,63]. The creatine phosphate shuttle is important in translocating ATP produced from oxidative phosphorylation in the mitochondrial to the cytosol and areas within the cell needing ATP for energy metabolism [50,56,57]. The creatine phosphate shuttle thereby serves as an important regulator of cellular metabolism. The role of creatine in energy metabolism and impact that creatine has on maintaining energy availability in diseases that depend on the CK/PCr system provides the metabolic basis on how creatine can affect health, disease, and provide therapeutic benefit [6,9,21,41,50,64,65,66,67,68,69,70,71]. The role of creatine in energy metabolism will be discussed in greater detail in another paper in this special issue.

## 4. General Health Benefits

Most creatine research initially focused on creatine’s role in exercise performance, training adaptations, and safety in untrained and trained healthy individuals [1]. Creatine supplementation has been reported to increase muscle creatine and PCr levels, enhance acute exercise capacity, and improve training adaptations [44,66,69,72,73,74,75,76,77,78,79,80,81,82,83,84,85,86,87,88,89,90,91,92,93,94,95,96]. The improvement in performance has generally been 10–20% on various high-intensity exercise tasks [97] that include lifetime fitness activities like fitness/weight training [77,84,91,98,99,100,101,102,103,104,105,106,107,108], golf [109], volleyball [110], soccer [82,111,112], softball [113], ice hockey [114], running [115,116,117,118,119], and swimming [73,74,120,121,122,123], among others. Ergogenic benefits have been reported in men and women from children to elderly populations, although the majority of studies have been conducted on men [74,111,113,124,125,126,127,128]. After comprehensively reviewing the literature, the International Society of Sports Nutrition (ISSN) concluded that creatine is “the most effective ergogenic nutritional supplement currently available to athletes in terms of increasing high-intensity exercise capacity and lean body mass during training” [1,42,44,89]. The American Dietetic Association, Dietitians of Canada, and the American College of Sports Medicine have come to similar conclusions in their position stands [129,130]. Thus, there is a strong scientific consensus that creatine supplementation is an effective ergogenic nutrient for athletes as well as individuals starting a health and fitness program.

As performance-related studies assessed health and safety markers, evidence began to accumulate that creatine supplementation may also offer some health and/or therapeutic benefits as we age [4,12,14,67,69,70,71,131]. In this regard, creatine supplementation has been reported to help lower cholesterol, triglycerides and/or manage blood lipid levels [77,132,133]; reduce the accumulation of fat on the liver [133,134]; decrease homocysteine thereby reducing risk of heart disease [30,135]; serve as an antioxidant [30,136,137,138,139]; enhance glycemic control [1,11,140,141,142,143]; reduce the progress of some forms of cancer [8,17,18,135,144,145,146,147]; increase strength and muscle mass [2,9,13,67,70,71,93,99,101,148,149,150,151,152,153,154]; minimize bone loss in some studies [2,4,14,16,99,150,155,156,157,158,159,160]; improve functional capacity in osteoarthritic and fibromyalgia patients [22,161,162]; enhance cognitive function particularly in older populations [3,27,28,69,94,127,131,159,163,164,165,166,167,168]; and, in some instances, improve the efficacy of some anti-depressant medications [5,29,169,170,171,172]. These findings support contentions that it is prudent for individuals to consume at least 3 g/day of creatine to support general health as one ages [1,50]. Therefore, although more research is needed, it can be reasonably concluded based on current evidence that creatine supplementation can increase cellular energy availability and support general health, fitness, and well-being throughout the lifespan.

## 5. Role of Creatine in Aging Populations

Several studies have evaluated the effects of creatine supplementation in older populations in an attempt to prevent sarcopenia, maintain strength, and/or reduce the risk of chronic disease. The following discusses some of these potential applications.

### 5.1. Muscle Mass, Strength, Bone and Body Composition

Sarcopenia is an age-related muscle condition characterized by a reduction in muscle quantity, muscle strength, and functional capacity. Although multifactorial, sarcopenia may be caused by changes in muscle protein kinetics (synthesis and breakdown), neuromuscular function, inflammation, physical activity, and nutrition [12,14]. We also generally lose strength, muscle mass, bone mass, balance while increasing body fat as we age, whether clinically diagnosed with sarcopenia or not [3,69,131]. A number of nutritional and exercise interventions have been suggested to counteract sarcopenia in older individuals, including creatine supplementation during resistance training [12,14]. For example, Brose and colleagues [173] were among the first to report that creatine supplementation (5 g/day for 14 weeks) during heavy resistance training promoted greater gains in muscle mass and isometric muscle strength in older adults (>65 years). Chrusch and coworkers [106] reported that older participants (60–84 years) who supplemented their diet with creatine (0.3 g/kg/day for 5 days and 0.07 g/kg/day for 79 days) during supervised resistance training (3 days/week for 12 weeks) experienced greater gains in lean tissue mass, lower-body maximal strength, and endurance, and isokinetic knee flexion/extension power compared to controls. Candow and colleagues [99] reported that creatine (0.1 g/kg/day) and protein (0.3 g/kg/day) supplementation increased muscle mass and strength while decreasing protein degradation and bone resorption markers in older men. Chilibeck and associates [150] found that creatine supplementation (0.1 g/kg/day) during 12 months of resistance training increased strength and bone density in postmenopausal women. Gualano and coworkers [98] reported that creatine supplementation (20 g/day for 5 days; 5 g/day for 161 days) during resistance training improved appendicular lean mass and muscle function in older vulnerable women and that creatine supplementation alone resulted in similar gains in muscle mass compared to those engaged in resistance training alone. Aguiar and coworkers [96] also found that creatine supplementation (5 g/day for 12 weeks) combined with resistance training improved muscle endurance, ability to perform functional tasks, maximal strength, and muscle mass in older women.

Additionally, McMorris et al. [174] reported that creatine supplementation (20 g/day for 7 days) after sleep deprivation improved balance measures. Bernat and colleagues [175] reported that creatine supplementation (0.1 g/kg/day) during 8 weeks of high-velocity resistance training in untrained healthy aging men promoted significantly greater gains in leg press and total lower-body strength, muscle thickness, and some measures of peak torque and physical performance. Moreover, a meta-analysis revealed that older individuals participating in resistance training experienced greater gains in muscle mass, strength, and functional capacity when supplementing their diet with creatine [91]. A similar meta-analysis conducted by Candow and colleagues [9] found that older individuals who took creatine during resistance training experienced significantly greater gains in muscle mass and upper body. While not all studies report statistically significant effects, the preponderance of available research supports contentions that creatine supplementation, when combined with resistance exercise, can help maintain or increase muscle mass, strength, and balance in older individuals and therefore serve as an effective countermeasure to attenuate sarcopenia. The role of creatine supplementation during resistance training in sarcopenic populations will be discussed in more detail in this paper series on aging, sarcopenia, and bone health.

In addition, people often experience adult-onset obesity as they age, prompting them to diet to promote weight loss. Unfortunately, this often leads to loss of muscle mass and strength, which would be counterproductive in older individuals. Creatine supplementation while following an energy-restricted diet may be an effective strategy to maintain muscle mass, promote fat loss, and help manage adult-onset obesity. In support of this contention, Forbes and colleagues [176] recently conducted a meta-analysis on the effects of creatine on body composition and found that creatine supplementation may not only help maintain muscle mass but also promote fat mass loss. This strategy could be helpful in preventing or managing adult-onset obesity. Thus, although more research is needed, it can be reasonably concluded based on available literature that creatine supplementation, particularly when combined with resistance training, can promote gains in strength and help maintain or increase muscle mass and bone density in older individuals. Further, creatine supplementation during energy-restriction-induced weight loss interventions may be an effective way to preserve muscle mass, promote fat loss, and thereby help manage adult-onset obesity.

### 5.2. Cognitive Function

Creatine supplementation has been reported to increase brain PCr content by 5–15% and thereby enhance brain bioenergetics [21,53,69,131,171]. Consequently, research has examined whether creatine supplementation affects cognition, memory, and/or executive function in older individuals as well as patients with mild cognitive impairment [94,168,174,177,178]. Several studies have found that creatine supplementation attenuates mental fatigue [27,28,127] and/or can improve cognition, executive function, and/or memory [28,94,127,168,177,179]. For example, Watanabe and associates [180] found that creatine supplementation (8 g/day for 5 days) increased oxygen utilization in the brain and reduced mental fatigue in participants performing repetitive mathematical calculations. Rae et al. [177] found that working memory and processing speed increased with creatine supplementation (5 g/day for 6 weeks). McMorris and colleagues [174] reported that sleep-deprived participants better maintained random movement generation, time to react to choices, mood state, and balance when supplemented with creatine (20 g/day for 7 days). These researchers also reported that random number generation, forward spatial recall, and long-term memory tasks were significantly improved in elderly participants when supplemented with creatine. Ling et al. [178] also reported that cognition on some tasks was improved with creatine ethyl ester supplementation (5 g/day for 15 days). More recently, VAN Cutsem and coworkers [27] reported that creatine supplementation (20 g/day for 7 days) prior to performing a simulated soccer match improved muscular endurance and prolonged cognitive performance. While more research is needed and not all studies show benefit [127,167], it can be reasonably concluded based on current scientific evidence that creatine supplementation may increase brain creatine content and/or support cognitive function, particularly as one ages.

### 5.3. Glucose Management and Diabetes

Creatine uptake into tissue is influenced by glucose and insulin [142,181,182]. Creatine supplementation has also been reported to prevent declines in the GLUT-4 transporter during immobilization while increasing GLUT-4 by 40% during rehabilitation after atrophy [140]. Moreover, co-ingestion of creatine with carbohydrate [47,183] or creatine with carbohydrate and protein [184] has been reported to increase creatine uptake and/or muscle glycogen levels [47,184,185]. Consequently, research has evaluated whether creatine supplementation may influence glucose management [10,11,140,141,142,143]. For example, Gualano et al. [141] evaluated the effects of creatine supplementation (5 g/day for 12 weeks) during training in participants with type 2 diabetes. The researchers found that creatine supplementation improved glucose tolerance to ingesting a standard meal, increased GLUT-4 translocation, and promoted a significant reduction in HbA1c levels. Moreover, the AMPK-alpha protein content tended to be higher after Cr supplementation and was significantly related to the changes in GLUT-4 translocation and Hb1Ac levels, suggesting that AMPK signaling may be implicated in the effects of supplementation on glucose uptake in type 2 diabetes [143]. Thus, there is evidence to suggest that creatine supplementation enhances glucose uptake and insulin sensitivity and, therefore, can help individuals manage glucose and HbA1c levels, particularly when initiating an exercise program [10,11,186]. Based on this literature, it can be reasonably concluded that **c**reatine supplementation may support healthy glucose management.

### 5.4. Heart Disease

Coronary artery disease limits blood supply to the heart, thereby increasing susceptibility to ischemic events, arrhythmias, and/or heart failure. Creatine and PCr play an important role in maintaining myocardial bioenergetics during ischemic events [21]. For this reason, there has been interest in assessing the role of creatine or PCr administration in reducing arrhythmias, ischemia-related damage, and/or heart function in individuals with chronic heart failure [187,188,189,190,191,192,193,194,195,196,197]. For example, Anyukhovsky et al. [195] reported that intravenous administration of PCr and phosphocreatinine (300 mg/kg) in canines prevented the accumulation of lysophosphoglycerides in the ischemic zone of the heart, which is associated with an increased prevalence of arrhythmias. The researchers concluded that this might explain the antiarrhythmic action of PCr and phosphocreatinine in acute myocardial ischemia. Sharov and coworkers [194] reported that exogenous PCr administration protected against ischemia in the heart. Likewise, Balestrino and coworkers [21] evaluated the effects of adding PCr to cardioplegic solutions on energy availability during myocardial ischemia. The researchers found that PCr administration improved energy availability to the heart, reduced the incidence of arrhythmias, and improved myocardial function. As noted below, there is also evidence that creatine supplementation may maintain energy availability during brain ischemia and reduce stroke-related damage. Moreover, several studies have reported some benefit of oral creatine supplementation in heart failure patients participating in rehabilitation programs [198,199,200,201]. While not all studies report benefit from oral creatine supplementation [23,202] and more research is needed, current evidence suggests that phospho**c**reatine administration and possibly creatine supplementation support heart metabolism and health, particularly during ischemic challenges.

## 6. Potential Therapeutic Role of Creatine Supplementation

Given the metabolic role of creatine and the PCR/CK system, particularly during ischemia and in some disease states, there has been interest in examining the potential therapeutic role of creatine in a number of clinical populations. The following provides a brief overview of some of this work as an introduction to topics that will be reviewed in greater detail in other papers in this special issue.

### 6.1. Creatine Synthesis Deficiencies

Some individuals are born with rare deficiencies in creatine-related enzymes or transporters (e.g., AGAT, GAMT, and CRTR) that reduce the ability to transport creatine into the cell or synthesize creatine endogenously [203]. There is also recent evidence that the human genome encodes 19 genes of the solute carrier 6 (SLC6) family and that non-synonymous changes in the coding sequence give rise to mutated or misfolded transporters that cause diseases in affected individuals [204]. This includes the creatine transporter (CT1, SLC6A8) in which deficiencies have been reported to account for about 2% of intellectual disabilities in boys [205]. Individuals with creatine synthesis deficiencies and creatine transporter mutations typically present with low brain Cr and PCr levels [53,61,204,206,207,208,209,210]. Low brain creatine content has been associated with muscle myopathies (e.g., weakness), voluntary or involuntary movement disorders that can affect muscle function and coordination, speech development, epilepsy, cognitive and motor development delays, and/or autism [53,61,203,204,206,207,208,209,210]. Individuals with these conditions have a greater dependence on dietary creatine. For this reason, high-dose, long-term creatine supplementation (e.g., 0.3–0.8 g/kg/day) throughout the lifespan is a nutritional strategy of increasing brain creatine content in these populations [53,61,203,204,206,207,208,209,210,211,212,213,214]. This research has generally found that long-term creatine supplementation can improve clinical outcomes, particularly in patients with AGAT and GAMT deficiencies [207].

For example, Bianchi et al. [215] found that creatine supplementation (200–800 mg/kg/day divided into 5 servings per day) significantly increased brain creatine and PCr levels in patients with GAMT-d and AGAT-d creatine synthesis deficiencies. Battini et al. [216] reported that a patient diagnosed at birth with AGAT deficiency who was treated with creatine supplementation beginning at four months of age experienced normal psychomotor development at eighteen months compared to siblings who did not have the deficiency. Stockler-Ipsiroglu and coworkers [217] evaluated the effects of creatine monohydrate supplementation (0.3–0.8 g/kg/day) in 48 children with GMAT deficiency with clinical manifestations of global developmental delay/intellectual disability (DD/ID) with speech/language delay and behavioral problems (*n* = 44), epilepsy (*n* = 35), or movement disorder (*n* = 13). The median age at treatment was 25.5 months, 39 months, and 11 years in patients with mild, moderate, and severe DD/ID, respectively. The researchers found that creatine supplementation increased brain creatine levels and improved or stabilized clinical symptoms. Moreover, four patients treated younger than nine months had normal or almost normal developmental outcomes. Long-term creatine supplementation has also been used to treat patients with ornithine aminotransferase (OAT) deficiency that causes gyrate atrophy of the choroid and retina due to secondary creatine depletion that is characterized by progressive vision loss [218,219,220,221,222]. These findings and others provide promise that high-dose creatine monohydrate supplementation is well tolerated and may be an effective adjunctive therapy for infants, children, and adults, particularly with AGAT deficiency [207,223,224,225,226]. Thus, it can be reasonably concluded that long-term, high-dose creatine supplementation in individuals with creatine synthesis can increase brain creatine and PCr levels and reduce the severity of deficits associated with these disorders.

### 6.2. Neurodegenerative Diseases and Muscular Dystrophy

Several studies have investigated the short- and long-term therapeutic benefit of creatine supplementation in animals, children, and adults with various neuromuscular diseases like Huntington’s disease (HD) [51,227,228,229,230,231,232]; Parkinson’s disease (PD) [51,66,100,227,233,234,235]; mitochondria-related diseases [58,235,236,237,238,239]; amyotrophic lateral sclerosis (ALS) [227,240,241,242,243,244,245,246]; spinal and bulbar muscular atrophy [247]; and, muscular dystrophies (MD) [248,249,250,251,252,253]. Several of these investigations, particularly in animal models, reported improved exercise tolerance and/or clinical outcomes. However, a large multi-site clinical trial conducted by Bender and coworkers [51] on PD, HD, and ALS patients did not find promising results. In this regard, they monitored 1687 participants who supplemented their diet with creatine (9.5 g/day for up to 5 years). The researchers did not observe statistically significant improvement in PD or ALS patient outcomes. However, in patients with HD, there was some evidence that creatine supplementation attenuated brain atrophy, suggesting some potential clinical benefit in this population. The reason animal studies may have yielded more promising results may be due to the fact that people typically do not present with symptoms of neurodegenerative disorders (e.g., ALS, HD, PD, etc.) until they have lost 70% or more of their alpha neurons. On the other hand, results in muscular dystrophy populations have been more promising because the muscle is the primary target. To support this contention, Kley and coworkers [254] conducted a Cochrane systemic review of the literature and found that high-quality evidence from randomized clinical trials (RCTs) demonstrated that short- and medium-term creatine supplementation increases muscle strength in muscular dystrophies and functional performance in muscular dystrophy and idiopathic inflammatory myopathy. However, assessment of high quality RCTs found no significant improvement in muscle strength in metabolic myopathies [254]. Thus, while creatine supplementation has been shown to have neuroprotective properties and improve muscle strength and endurance in patient populations, the efficacy of long-term, high-dose creatine supplementation in individuals with neurodegenerative diseases is currently equivocal, while promising, in patients with muscular dystrophy.

### 6.3. Brain and Spinal Cord Neuroprotection

It is well known that creatine supplementation increases brain bioenergetics [21,166,215,235,255,256] and has neuroprotective benefits, particularly in response to injury and/or ischemic conditions [58,64,66,257]. Consequently, there has been interest in determining the effects of creatine supplementation on cerebral ischemia, stroke, traumatic brain injury (TBI), and spinal cord injury (SCI). For example, Adcock and associates [258] prophylactically administered neonatal rats creatine (3 g/kg for 3 days) and assessed brain bioenergetics in response to a cerebral ischemic event. The researchers found that creatine feeding significantly increased the ratio of brain PCr to Pi and promoted a 25% reduction in the volume of brain damage. Prass and coworkers [259] found that creatine administration decreased ischemia-induced brain infarction size by 40%. Zhu and colleagues [260] reported that oral creatine feeding in mice decreased the size of ischemia-induced brain damage and attenuated neuronal cell death, thereby providing neuroprotection. Allah and colleagues [261] found that neonatal mice fed creatine monohydrate for 10 weeks experience less ischemia-induced brain damage, as well as had better learning/memory during recovery. Finally, Turner and coworkers [166] reported that 7 days of creatine supplementation increased brain creatine content by 9.2%, increased corticomotor excitability, and prevented the decline in attention during hypoxia in healthy adults. Collectively, these findings suggest that prophylactic creatine supplementation may reduce the severity of brain ischemia and therefore may have some therapeutic benefits in individuals at risk to stroke [8,21,197].

Several studies have also evaluated the impact of creatine supplementation on mild traumatic brain injury (TBI) and spinal cord injury (SCI) outcomes in animals [3,6,171,262,263,264,265,266]. For example, Sullivan and coworkers [264] found that provision of creatine in the diet for 5 days prior to TBI decreased the amount of cortical brain damage by 36% in rats and 50% in mice. The researchers attributed the reduction in cortical damage to an improved energy availability. Hausmann and associates [265] reported that rats fed creatine (5 g/100 g dry food) prior to and following moderate SCI experienced less scar tissue and improved locomotor function test performance compared to controls. Moreover, Rabchevsky et al. [267] reported that rats fed a diet with 2% creatine for 4–5 weeks prior to and following SCI experienced less loss of gray matter. While these types of studies could not be performed in humans, they support contentions that creatine supplementation may reduce the severity of TBI and/or SCI. In humans, creatine supplementation has also been reported to enhance training adaptations in patients recovering from SCI. For example, Jacobs et al. [268] reported that creatine supplementation (20 g/day for 7 days) enhanced aerobic exercise capacity and ventilatory anaerobic threshold in patients with cervical SCI. Moreover, Amorim et al. [266] reported that individuals with SCI who consumed creatine (3 g/day for 8 weeks) with vitamin D (25,000 IU/day) while participating in a resistance-training program experienced significantly greater improvements in arm muscle area, strength, and functional capacity. While some studies report no benefit of creatine supplementation in patients with SCI [269,270], there is compelling evidence that creatine supplementation may reduce the severity of mild concussions, TBI, and/or SCI in animal models [21,263]. In fact, this evidence was so strong that the International Society of Sports Nutrition recommended that all athletes who are involved in sports with risk to TBI and/or SCI should take creatine to reduce the severity of these types of injury [1]. Based on this literature, it can be reasonably concluded that **c**reatine supplementation can enhance energy availability during ischemic events and provide neuroprotection from TBI and/or SCI.

### 6.4. Enhanced Rehabilitation Outcomes

Since creatine supplementation has been reported to increase resistance-training adaptations, a number of studies have examined whether creatine supplementation may enhance physical therapy outcomes from musculoskeletal injury [25,159,171,247]. For example, Hespel and associates [26] reported that creatine supplementation (20 g/day and reduced to 5 g/day during immobilization, 15 g/day during the first 3 weeks of rehabilitation, and 5 g/day for the remaining 7 weeks) promoted increases in myogenic regulating factor 4 (MRF4) and myogenic protein expression, which was associated with greater muscle fiber area (+10%) and peak strength (+25%) during rehabilitation. Jacobs et al. [268] reported that creatine supplementation (20 g/d for 7 days) increased peak oxygen uptake and ventilatory anaerobic threshold in patients with cervical-level spinal cord injury (SCI). Moreover, several studies reported that creatine supplementation in chronic heart failure and chronic obstructive pulmonary disease (COPD) patients enhanced rehabilitative outcomes [23,198,199,200,202,271,272,273]. For example, Andrews and colleagues [199] found that creatine supplementation (20 g/day for 5 days) in chronic heart failure patients augmented skeletal muscle endurance and attenuated the abnormal skeletal muscle metabolic response to exercise. Fuld et al. [271] reported that creatine supplementation (17.1 g/day for 2 weeks prior to rehabilitation and 5.7 g/day for 16 weeks during rehabilitation) increased fat-free mass, peripheral muscle strength, and endurance, and health status in patients with COPD. Hass and colleagues [100] reported that creatine supplementation (20 g/day for 5 days and 5 g/day for 12 weeks) during resistance training in PD patients promoted greater muscle strength and ability to perform the functional chair sit-to-rise test. Cooke and assistants [274] reported that creatine supplementation prior to (0.3 g/kg/day for 5 days) and following (0.1 g/kg/day for 14 days) performing an eccentric-resistance-only exercise bout designed to promote muscle injury significantly reduced markers of muscle damage and hastened recovery of muscle function. Finally, Neves et al. [22] reported that creatine supplementation (20 g/day for 5 days and 5 g/day for 79 days) improved physical function, lower-limb lean mass, and quality of life in postmenopausal women with knee osteoarthritis undergoing strengthening exercises. Conversely, some studies have found no statistically significant effects of creatine supplementation during recovery from orthopedic injury. For example, Roy et al. [275] reported that creatine supplementation (10 g/day for 10 days before surgery and 5 g/day for 30 days after surgery) did not improve body composition, muscle strength, or enhance recovery in osteoarthritic patients who underwent total knee arthroplasty. Likewise, Tyler et al. [276] reported that creatine supplementation (20 g/day for 1 week and 5 g/day for 11 weeks) after anterior cruciate ligament (ACL) reconstruction had no significant effects on isokinetic strength measures during or following rehabilitation. Although more research is needed, there is evidence that creatine supplementation prior to and following injury may reduce immobilization-related atrophy and/or enhance rehabilitative outcomes in a number of populations.

### 6.5. Pregnancy

Since creatine supplementation has been shown to improve cellular bioenergetics during ischemic conditions and possess neuroprotective properties, there has been interest in creatine use during pregnancy to promote neural development and reduce complications resulting from birth asphyxia [7,277,278,279,280,281,282,283,284,285]. The rationale for creatine supplementation during pregnancy is that the fetus relies upon placental transfer of maternal creatine until late in pregnancy, and significant changes in creatine synthesis and excretion occur as pregnancy progresses [7,280]. Consequently, there is an increased demand for and utilization of creatine during pregnancy. Maternal creatine supplementation has been reported to improve neonatal survival and organ function following birth asphyxia in animals [277,278,279,281,282,283,285]. In humans, there is evidence that the creatine needs of the mother increase during pregnancy [7,280]. Consequently, there has been interest in determining the role of creatine during pregnancy on fetal growth, development, and health of the mother and child [7,280,286,287,288]. Available literature suggests that creatine metabolism may play an essential role in the bioenergetics of successful reproduction and that creatine supplementation may improve reproductive and/or perinatal outcomes [7,277,278,279,280,283,284,286,288]. However, it should be noted that research on the role of creatine supplementation in pregnant women is limited. While creatine supplementation has been reported to be safe in a number of populations [10,42,171,289,290] and there is no evidence that creatine supplementation poses a risk for women of reproductive age or preterm infants [287,288,291], additional safety and tolerability studies in pregnant women and those trying to conceive are needed. Consequently, although there is emerging evidence that creatine supplementation may help support the mother and child’s nutritional needs and health, due to the limited studies in pregnant humans, caution should be exercised when recommending use during human pregnancy.

### 6.6. Immune Support

One of the more novel potential uses of creatine is its influence on the immune system. A number of in vitro and animal studies indicate that creatine has immunomodulatory effects [6]. In this regard, several studies have reported that creatine supplementation may alter production and/or the expression of molecules involved in recognizing infections like toll-like receptors (TLR) [6]. For example, Leland and colleagues [292] reported that creatine down-regulated expression of TLR-2, TLR-3, TLR-4, and TLR-7 in a mouse macrophage cell line (RAW 254.7). While this could reduce the ability to sense some infections in immunocompromised individuals, TLR-4 downregulation may also alter Parkinson’s disease pathology and inhibit neuronal death as the disease progresses [293,294]. There is also evidence that creatine influences cytokines possibly via the NF-κB signaling pathway, thereby affecting cytokines, receptors, and/or growth factors that can positively or negatively influence immune response [6,292]. A creatine-induced reduction of pro-inflammatory cytokines (e.g., IL-6) and other markers of inflammation (e.g., TNFα, PGE2) may help explain some of the neuroprotective benefits observed in patients with central nervous system-related diseases [6]. It may also explain reports that creatine supplementation attenuates inflammatory and/or muscle damage in response to intense exercise [274,295,296,297]. On the other hand, there have been several studies in mice suggesting that creatine supplementation may impair airway inflammation, thereby exacerbating exercise-induced asthma [298,299]. However, other studies suggest that creatine attenuates the pulmonary and systemic effects of lung ischemia in reperfusion injury in rats [300]; improves rehabilitative outcomes in patients with cystic fibrosis [301] and COPD [271]; or, has no statistically significant effects on pulmonary rehabilitation outcomes [24,273] and youth soccer players with allergies [302]. Additional research is needed to understand creatine’s anti-inflammatory and immunomodulating effects, but it is clear that creatine can affect these pathways. Thus, there is evidence to suggest that supplementation may have anti-inflammatory and immunomodulating effects.

### 6.7. Anticancer Properties

Another emerging area is related to the potential anticarcinogenic effects of creatine supplementation. As noted above, creatine and phosphagens play an important role in maintaining energy availability [38,39,56,57], particularly related to the role of the CK/PRr system and shuttling of ATP, ADP, and Pi in and out of the mitochondria for cellular metabolism [50,54,55]. Prior studies have shown that creatine content and energy availability are low in several types of malignant cells and T cells that mediate the immune responses against cancer [17,18,144,145,147]. Additionally, the creatine transport *SLC6A8* gene expression encodes a surface transporter controlling the uptake of creatine into a cell, markedly increases in tumor-infiltrating immune cells [17]. It has been well established that creatine and its related compound cyclocreatine have anticancer properties [144,303,304]. For example, Patra et al. [144] also noted that the efficacy of the anticancer medication methylglyoxal (MG) is significantly augmented in the presence of creatine and that administration of creatine, methylglyoxal, and ascorbic acid provided greater efficacy and eliminated visible signs of tumor growth. Moreover, creatine and CK, which were very low in sarcoma tissue, were significantly elevated with the concomitant regression of tumor cells. Similarly, Pal and colleagues [147] reported that MG efficacy was improved with co-administration of creatine and ascorbic acid in muscle cells in vitro and in sarcoma animal model in vivo, suggesting that creatine supplementation may serve as an adjunctive anticancer therapeutic intervention with MG. Di Biase and coworkers [17] also reported that creatine uptake deficiency severely impaired CD8 T cell responses to tumor challenge in vivo and to antigen stimulation in vitro, while supplementation of creatine through either direct administration or dietary supplementation significantly suppressed tumor growth in multiple mouse tumor models. Moreover, the energy-shuttling function of creatine goes beyond regulating CD8 T cells, in that reduced energy capacity has also been reported in multiple immune cells in various mouse tumor models in creatine transporter knockout mice [17]. The researchers concluded that creatine is an important metabolic regulator controlling antitumor T cell immunity and that creatine supplementation may improve T cell–based cancer immunotherapies [17]. Collectively, these findings indicate that creatine supplementation may have anticancer properties. Thus, it can be reasonably concluded based on available evidence that creatine is an important energy source for immune cells, can help support a healthy immune system, and may have some anticancer properties.

### 6.8. Improve Functional Capacity in Patients with Chronic Fatigue?

Chronic fatigue syndrome (CFS), also known as post-viral fatigue syndrome (PFS) or myalgic encephalomyelitis (ME), is characterized by fatigue and associated symptoms (e.g., muscle and joint pains, anxiety, cognitive and sleep disorders, intolerance to physical exertion) persisting more than six months in duration [305]. Although the etiology of these conditions are unknown, there has been some recent interest in whether creatine may help improve functional capacity and thereby help people with CFS conditions better manage this condition. Although controversial, there is some evidence that a lack of creatine availability and/or impaired creatine metabolism may play a role in CFS-related diseases. For example, Malatji et al. [306] reported a significant relationship between urinary creatine levels and symptoms of pain, fatigue, and energy levels in patients with CFS-related chronic pain syndrome, fibromyalgia. Mueller and associates [307] reported that creatine levels in the left parietal cortex was significantly lower in patients with ME/CFS, while higher in the left putamen and not affected in 45 other areas examined. Moreover, when using creatine as the denominator to normalize values, significant differences were observed in the ratio of N-acetylasparte/creatine, choline/creatine, lactate/creatine, and myo-inositol/creatine ratios between CFS and controls. In a similar study, van der Schaaf et al. [308] reported that greater pain levels inversely related to the N-acetylaspartylglutamate/creatine ratio in the dorsolateral prefrontal cortex of a group of 89 women with CF compared to controls. While it is unclear how changes in brain metabolites, including creatine, are involved in the pathology or symptomology of CFS, creatine and GAA supplementation have been reported to increase brain creatine content and might thereby help normalize some of these ratios. Although this is highly speculative and needs additional research, it is interesting to note that alterations in the ratio of brain metabolites to creatine have been implicated in CFS.

With that said, several studies have investigated the role of creatine or creatine-related compounds on patient outcomes in CFS patients. For example, Amital and coworkers [309] reported that creatine supplementation (3 g/day for 7 days and 5 g/day for 21 days) in a patient presenting with post-traumatic stress disorder, depression, and fibromyalgia showed improvement in symptoms of depression, pain measures, and quality of life. The patient continued supplementation for another 4 weeks and retained these benefits. Leader et al. [310] conducted an open-label study to assess the effects of creatine supplementation (3 g/day for 3 weeks and 5 g/day for 5 weeks) as an adjunctive nutritional therapy in 16 patients with Fibromyalgia Syndrome. The researchers found that creatine supplementation significantly improves markers related to the severity of fibromyalgia, disability, pain, sleep quality, and quality of life. The improvements observed returned toward baseline after 4 weeks after stopping creatine therapy. Alves and colleagues [162] reported that creatine supplementation (20 g/day for 5 days; 5 g/day for 107 days) increased intramuscular phosphorylcreatine content and improved lower- and upper-body muscle function, with minor changes in other fibromyalgia features. The authors concluded that creatine supplementation may serve as a useful dietary intervention to improve fibromyalgia patients’ muscle function. Finally, Ostojic and colleagues [311] reported that GAA supplementation (2.4 g/day for 3 months) positively affected creatine metabolism and work capacity in women with CFS but did not affect general fatigue symptoms musculoskeletal soreness. While all studies do not report benefits, these findings provide some support that creatine and/or GAA may have some therapeutic benefit for patients with CFS, PFS, ME, and/or fibromyalgia. However, it should be noted that the improvements in functional capacity observed in these studies are similar to those observed in healthy individuals who take creatine and that pain indices were not significantly affected in all of these studies. Nevertheless, although more research is needed, it can be reasonably concluded that creatine and/or GAA may improve functional capacity in patients with chronic fatigue-related syndromes such as post-viral fatigue syndrome (PFS) and myalgic encephalomyelitis (ME).

### 6.9. Antidepressive Effects

Reports since the early 1980s have suggested that creatine metabolism and/or availability may have antidepressive effects [312,313,314,315,316,317,318]. These studies and others have provided the basis for assessing the effects of creatine and/or creatine precursors like S-adenosyl-L-methionine (SAMe) and GAA affect brain phosphagen levels, markers of depression, and/or the therapeutic efficacy of antidepressant medications [8,169,170]. For example, the creatine precursor SAMe has been reported to be an effective treatment for clinical depression. Silveri et al. [316] reported that SAMe supplementation (1600 mg/day) increased brain creatine and PCr levels and lowered transverse relaxation time (T2RT) using magnetic resonance spectroscopy (^31^P MRS) in nondepressed subjects; this effect was larger in women compared to men. Allen and colleagues [319] reported that rats fed creatine diets (4%) for 5 weeks altered depression-like behavior in response to forced swim training in a sex-dependent manner, with female rats displaying an antidepressant-like response. Ahn and coworkers [320] reported that a single treatment of creatine or exercise has partial effects as an antidepressant in mice with chronic mild stress-induced depression and that combining creatine and exercise promoted greater benefits. Pazini et al. [321] reported that creatine administration (21 days, 10 mg/kg, p.o.) abolished corticosterone-induced depressive-like behaviors in mice. Similarly, Leem and colleagues [322] reported that mice exposed to mild chronic stress for 4 weeks had a greater effect on hippocampal neurogenesis via the Wnt/GSK3beta/beta-catenin pathway activation when creatine and exercise were combined compared with each treatment in chronic mild stress-induced behavioral depression. There is some support in human trials that creatine supplementation may affect depression [171,323]. For example, Bakian et al. [324] recently assessed the dietary patterns from the National Health and Nutrition Examination Survey (NHANES) database and found a significant negative relationship between dietary creatine intake and depression among adults in the United States. Roitman et al. [169] reported in an open-label study that creatine monohydrate supplementation (3–5 g/day for 4 weeks) improved outcomes in a small sample of patients with unipolar depression. Toniolo et al. [29] evaluated the effects of creatine supplementation (6 g/day for 6 weeks) in bipolar patients and reported on Montgomery–Asberg Depression Rating Scale (MADRS) remission rates (i.e., 66.7% remission in the creatine group vs. 18.2% in the placebo group). In a similar study [29], this group reported that adjunctive creatine therapy (6 g/day for 6 weeks) in patients with bipolar depression improved verbal fluency tests. Moreover, in a proof-of-concept study [172], these researchers reported that creatine supplementation (6 g/day for 6 weeks) in patients with bipolar disorder type I or II enhanced remission MADRS scores in participants who completed the study. Although more research is needed, there is some evidence suggesting that creatine may help individuals manage some types of depression and/or anxiety disorders, particularly when combined with choline [325,326]. Thus, there is evidence that creatine supplementation may support mental health.

### 6.10. Fertility

Since sperm motility is dependent on ATP availability and CK activity has been associated with greater sperm quality and function [50,327,328,329], there has been some interest in whether creatine supplementation and/or administration might improve fertility. For example, creatine has been added to medium during intrauterine insemination to increase the viability of sperm and the success of fertility treatments [327,328,329,330,331,332]. Although more research is needed, these findings suggest that creatine may play an important role in fertility and support reproductive health.

### 6.11. Skin Health

Since creatine availability has been reported to affect energy status in the dermis and is an antioxidant, several studies have evaluated whether creatine’s topical application influences skin health and/or may serve as an effective anti-wrinkle intervention [333]. For example, Lenz et al. [333] reported that stress decreases CK activity in cutaneous cells and that topical creatine application improved cellular energy availability and markedly protected against a variety of cellular stress conditions, like oxidative and UV damage, which are involved in premature skin aging and skin damage. Peirano and coworkers [334] found that topically applied creatine rapidly penetrates the dermis, stimulates collagen synthesis, and influences gene expression and protein. Additionally, the topical application of a creatine-containing formulation for 6 weeks significantly reduced the sagging cheek intensity in the jowl area, crow’s feet wrinkles, and wrinkles under the eyes. The researchers concluded that creatine represents a beneficial active ingredient for topical use in the prevention and treatment of human skin aging. Thus, there is evidence that creatine supports skin health.

## 7. Conclusions

The benefits of creatine monohydrate supplementation go well beyond increasing muscle Cr and PCr levels and thereby enhancing high-intensity exercise and training adaptations. Research has clearly shown several health and/or potential therapeutic benefits as we age and in clinical populations that may benefit by enhancing Cr and PCr levels. Although additional research is needed to explore further the health and potential therapeutic benefits of creatine supplementation, many of these topics will be described in more detail in other papers within this special issue. Based on the available evidence, the following can be reasonably concluded based.

1. Creatine supplementation can increase cellular energy availability and support general health, fitness, and well-being throughout the lifespan.
2. Creatine supplementation, particularly with resistance training, can promote gains in strength and help maintain or increase muscle mass in older individuals. Additionally, creatine supplementation during energy-restriction-induced weight loss may be an effective way to preserve muscle while dieting and thereby help manage adult-onset obesity.
3. Creatine supplementation may support cognitive function, particularly as one ages.
4. Creatine supplementation may support healthy glucose management.
5. Phosphocreatine administration and possibly creatine supplementation may support heart metabolism and health, particularly during ischemic challenges.
6. Long-term, high-dose creatine supplementation in individuals with creatine synthesis deficiencies can increase brain creatine and PCr levels and may reduce the severity of deficits associated with these disorders.
7. Although creatine supplementation has been shown to have neuroprotective properties and improve strength and endurance, the efficacy of long-term, high-dose creatine supplementation in individuals with neurodegenerative diseases is equivocal, while promising, in patients with muscular dystrophy.
8. Creatine supplementation may increase brain creatine content, enhance energy availability during ischemic events, and provide neuroprotection from TBI and/or SCI.
9. Creatine supplementation prior to and following injury may reduce immobilization-related atrophy and/or enhance rehabilitative outcomes in a number of populations.
10. Creatine supplementation during pregnancy may help support the mother and child’s nutritional needs and health; however, due to the limited studies in pregnant humans, caution should be exercised when recommending use during human pregnancy.
11. Creatine supplementation may have anti-inflammatory and immunomodulating effects.
12. Creatine is an important energy source for immune cells, can help support a healthy immune system, and may have some anticancer properties.
13. Creatine and/or GAA may improve functional capacity in patients with chronic fatigue-related syndromes such as post-viral fatigue syndrome (PFS) and myalgic encephalomyelitis (ME).
14. Creatine may support mental health.
15. Creatine may support reproductive health.
16. Creatine may support skin health.

## Data Availability

Not applicable.
